# Advances in redox-responsive drug delivery systems of tumor microenvironment

**DOI:** 10.1186/s12951-018-0398-2

**Published:** 2018-09-22

**Authors:** Xiaoshuang Guo, Yuan Cheng, Xiaotian Zhao, Yanli Luo, Jianjun Chen, Wei-En Yuan

**Affiliations:** 1 Engineering Research Center of Cell & Therapeutic Antibody, Ministry of Education, and School of Pharmacy, Shanghai Jiao Tong University, No. 800 Dongchuan RD, Shanghai, 200240 China; 2 Department of Pathology, Shanghai Jiao Tong University Affiliated Sixth People’s Hospital, 600 Yi-Shan Road, Shanghai, 200233 People’s Republic of China; 3 School of Pharmaceutical Sciences, Guangdong Provincial Key Laboratory of New Drug Screening, Southern Medical University, Guangzhou, China

**Keywords:** Redox-responsive, Drug delivery system, Antitumor, Disulfide bond

## Abstract

With the improvement of nanotechnology and nanomaterials, redox-responsive delivery systems have been studied extensively in some critical areas, especially in the field of biomedicine. The system constructed by redox-responsive delivery can be much stable when in circulation. In addition, redox-responsive vectors can respond to the high intracellular level of glutathione and release the loaded cargoes rapidly, only if they reach the site of tumor tissue or targeted cells. Moreover, redox-responsive delivery systems are often applied to significantly improve drug concentrations in targeted cells, increase the therapeutic efficiency and reduce side effects or toxicity of primary drugs. In this review, we focused on the structures and types of current redox-responsive delivery systems and provided a comprehensive overview of relevant researches, in which the disulfide bond containing delivery systems are of the utmost discussion.

## Background

In tumor therapy, developing a safe and efficient drug delivery system is a key factor to the success. With the rapid development of nanotechnology and materials, nano-carriers have been gradually applied in drug and gene delivery [1]. Antitumor nano drug delivery systems (ATNDDS) have been widely studied in recent years [2]. ATNDDS can improve the solubility and stability of drugs, enhance the targeting ability, reduce potential toxicity, achieve the co-delivery of drugs and genes, and realize the collaborative treatment [3]. In addition, it was reported that ATNDDS could be developed to alleviate the problem of drug resistance in cancer therapy [3].

Tumor tissues show different cellular microenvironments (e.g. acidic environments, enzyme environments, and reducing environments) for their unique physiological characteristics [4]. Developing nano-carriers that could respond to these unique physiological microenvironments was found to be a good way to release cargoes rapidly after entering cells [5, 6]. When the concentration of cargos in cells reached a certain threshold, tumor cells could be effectively killed. In recent years, a great progress has been made in developing redox-responsive nano-carriers. This review provides a comprehensive overview in relevant researches, and focuses on the structures and types of redox-responsive delivery systems containing disulfide bonds.

## The reducing environment of tumor cells

The reducing environment of tumor cells is strictly controlled and mainly determined by the reduction and oxidation states of NADPH/NADP^+^ and glutathione (GSH, GSH/GSSG), both of which have different reduction potentials and capacities [7]. In a reducing environment, concentration of GSH is higher than that of NADPH, in which case GSH plays a major role in the regulation of the microenvironment. At molecular levels, GSH controls the cellular reducing environment mainly through the formation and fragmentation of disulfide bonds and the reaction with excess ROS. And this is why the concentration of GSH is typically regarded as a proxy for the cellular reducing environment [8, 9]. The intracellular concentration of GSH can reach 10 mM, while the extracellular concentration of that ranges from 2 to 20 μM [10]. It was reported that the concentration of GSH in tumor tissues was at least 4-fold higher than that in normal tissues, and was especially high in some multi-drug resistant tumors [11, 12]. Through these mechanisms, we could find that the differences in reducibility of the environments between normal and tumor cells provide a potentially feasible strategy for targeted therapy with the help of redox-responsive ATNDDS [13].

## Redox-responsive delivery systems

The reducing environment of tumors serve as a unique internal signal that allows redox-responsive nanocarriers to degrade in tumor cells and release loaded cargoes. There are mainly three advantages of redox-responsive nanocarriers. First, they are often stable in normal tissues, which can obviously reduce the biological toxicity and side effects of both carriers and cargoes. Second, they show a prompt response to high GSH concentration in tumor cells to release cargoes (usually a few minutes to hours). Finally, compared to other potential sites of cargo release, the release in cytoplasm is often expected to have better therapeutic effects [11, 14]. In this review, we summarized currently existing redox-responsive carriers into the following categories based on their differences in structure.

### Redox-responsive delivery systems with disulfide bonds

Redox-responsive delivery systems with disulfide bonds are well studied in many researches. Disulfide bonds can be easily broken down by reducing glutathione into sulfhydryl groups, which causes the degradation of carriers and facilitates the release of cargoes. The specific application of this kind of delivery system is described in details in the following context.

### Redox-responsive delivery systems with diselenide bonds

With many studies on the redox-responsiveness of disulfide bonds going on, diselenide bonds are attracting much attention as well. Diselenide bonds have similar reduction sensitivity and redox-responsive ability as that of disulfide bonds [15, 16]. As the Se–Se bond and C–Se bond are with lower bond energy than that of S–S bonds (Se–Se 172 kJ/mol; C–Se 244 kJ/mol; S–S 268 kJ/mol), a more sensitive redox-responsive delivery system can be designed with the use of diselenide bonds in tumor therapy [17, 18].

Gang Cheng et al. added the active ester containing diselenide bonds to the branched oligoethyleneimine 800 Da (OEI_800_) to synthesize the polycationic carrier OEI_800_-SeSe_x_ [19]. The results showed that OEI_800_-SeSe_x_ and OEI_800_-SS_x_ shared similar redox-responsive degradation properties, and their cytotoxicity was significantly lower than that of PEI_25k_. Besides that, the transfection efficiency of both OEI_800_-SeSe_x_ and OEI_800_-SS_x_ was significantly higher than that of OEI_800_. However, the selenium-containing system is still in its infancy owing to its poor solubility and stability, which remains an unsolved problem for existing synthetic methods [20].

### Other redox-responsive delivery systems

In addition to disulfide and diselenide bonds, there are still some studies on other redox-responsive chemical structures. For example, succinimide-thioether linkage is sensitive to reducing environments and can be cleaved by exogenous glutathione to achieve rapid intracellular release [21]. When compared to that of disulfide bonds, succinimide-thioether linkage showed a higher blood stability and slower cargo release.

Delivery systems with ‘‘trimethyl-locked’’ benzoquinone (TMBQ) are also sensitive to reducing environments, which were reported to deliver drugs to solid tumors [22, 23]. With the reduction of Na_2_S_2_O_4_, TMBQ is detached from the backbone, resulting in the degradation of nanoparticles. In vitro drug release experiments showed that this novel delivery system can release 52% of the drug in 3 h in the presence of Na_2_S_2_O_4_. However, the reduction concentration of sodium salicylate used in the experiments was relatively higher compared to the intracellular concentration of GSH. Therefore, the exact release capacity of delivery systems with TMBQ in tumor cells is still unclear and needs to be further explored in future studies.

## Application of disulfide bonds in redox-responsive delivery systems

**Fig. 1:**
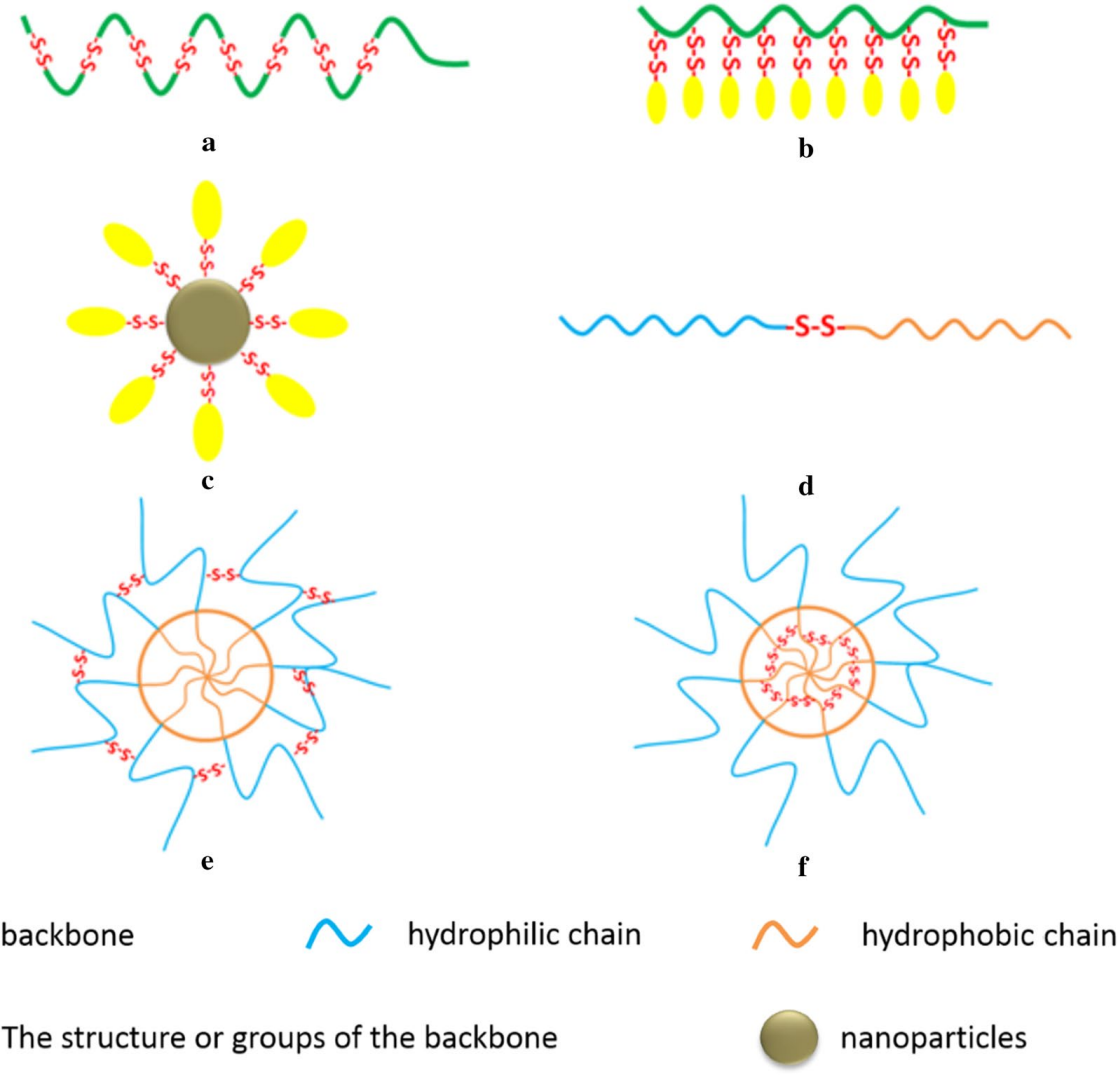
Schematic representation of disulfide bonds in redox-responsive delivery systems (Reprinted with permission from [20]. Copyright 2014 Royal Society of Chemistry) **a** Disulfide bonds are present in the backbone. **b** Disulfide bonds are present in side chains. **c** Disulfide bonds attached to the surface of nanoparticles. **d** Disulfide bonds link two moieties. **e** Shell crosslinked micelles. **f** Core crosslinked micelles

Delivery systems with disulfide bonds can be cleaved in high concentration of GSH, with which the carrier could degrade rapidly to release cargoes. Current applications of disulfide bonds in redox-responsive delivery systems are mainly compose of the use of disulfide bonds as linkers and cross-linking agents (as shown in Fig. 1) [20].

### Disulfide bonds acting as linkers

Disulfide bonds are often used in delivery systems as linkers, which can degrade rapidly to release cargoes in the reducing environment of GSH in tumor cells. This responsive bond can be attached to the backbone of polymers and used in the conjugation of side chains or nanoparticles with drugs, genes, and targeting groups.

#### Disulfide bonds present in the backbone

In the GSH reducing environment, carriers with disulfide bonds in the backbone (Fig. 1a) are depolymerized at a faster rate than other types of redox-responsive carriers. The delivery system with disulfide bonds in the backbone can be further divided to two categories: monomers containing disulfide bonds and monomers containing sulfhydryl groups. However, the stability of this structure (disulfide bond) is not as good as other categories, and it is difficult to be modified structurally. Therefore, this type of carriers with high content of disulfide bonds were studied less extensively.

**Fig. 2:**
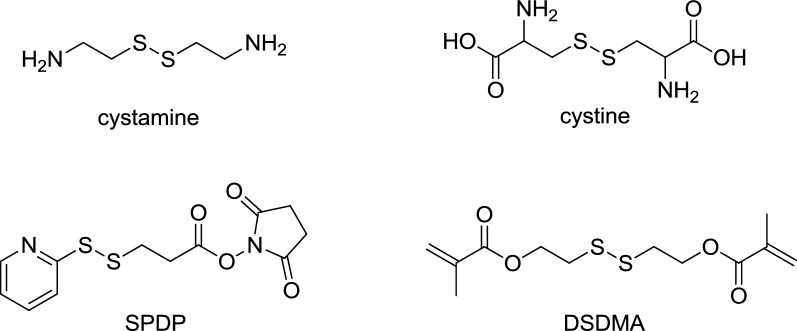
Disulfide-containing fragments commonly used in polymers

**Fig. 3:**
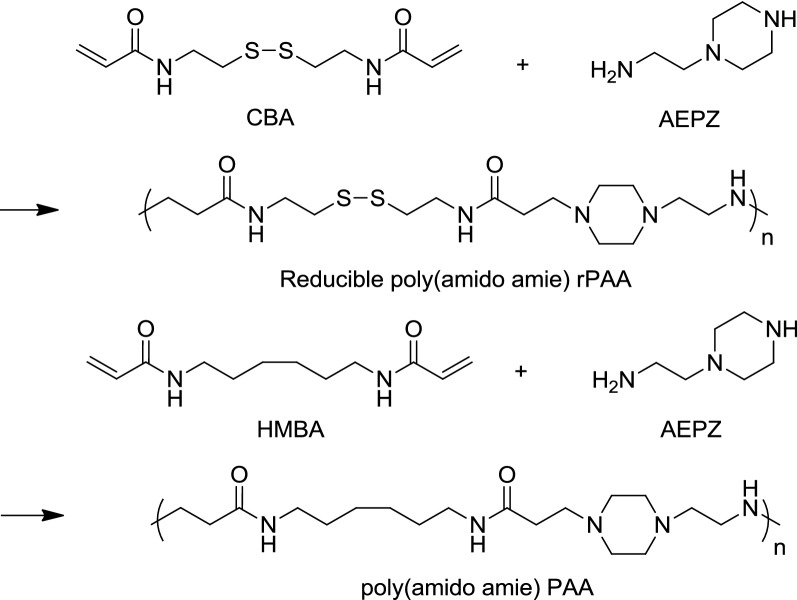
Synthetic scheme of rPAA and PAA (Reprinted with permission from [28]. Copyright 2010 Elsevier)

Polymeric carriers synthesized from disulfide-containing monomers are composed of many repeating units, which means the disulfide bond occupies a relatively high proportion throughout the polymer system. Therefore, when encountering the reducing environment, the polymer is with a high degree of response and degrades, after which cargoes will be released completely. Disulfide-containing fragments commonly used in polymers include cystamine [24], cystine [25], SPDP (*N*-succinimidyl-3-(2-pyridyldithiol) propionate) [26], DSDMA (disulfide-based dimethacrylate) [27] and other structures (Fig. 2). For example, David Oupický and his co-workers utilized *N,N′*-cystaminebisacrylamide (CBZ) and 1-(2-Aminoethyl) piperazine (AEPZ) as raw materials to synthesize reducible PAA (rPAA) (Fig. 3) [28]. And then they compared rPAA with poly(amido amines) (PAA) in cellular experiments, which share similar structures. It was demonstrated that in all tested cell lines, rPAA with high content of disulfide bonds is less cytotoxic than PAA, and rPAA released cargoes at a faster rate and can silence the luciferase gene more rapidly.

Due to the immaturity of mercapto polymerization, there are few researches about this method currently. Therefore, existing delivery systems that are based on the mercapto polymerization are very rare. There was a study utilizing mercapto polymerization to modify spermine with thiolate, and a gene carrier named bioreducible polyspermine (BPS) with linear structures was successfully synthesized [29]. Different BPS with various molecular weight (27.5 kDa, 15.7 kDa and 6.1 kDa) were analyzed in this study. It was found that the cytotoxicity of all BPS was significantly lower, and they were with higher transfection efficiency, compared to that of PEI 25k (control group).

#### Disulfide bonds present in side chains

Disulfide bonds present in side chains are usually used to modify the main chain (Fig. 1b). Attaching targeting groups to the backbone can increase the selectivity of carriers. Hydrophobic backbone connecting hydrophilic structures [30] or hydrophilic backbone connecting hydrophobic structures [31, 32] can promote the formation of micelles. In addition, side chains can also be linked to drugs to promote drug delivery [33, 34]. Redox-responsive delivery systems with disulfide bonds present in side chains are more multi-functional and easier to be chemically modified compared to those with disulfide bonds in the backbone.

A study utilized progressive ring-opening reactions of polysuccinimide (PSI) to prepare a redox and pH dual stimuli-responsive poly(aspartic acid) derivative to control the release of drugs [30]. Polyaspartamide backbone grafted polyethylene glycol (PEG) chains through redox-responsive disulfide bonds, forming a sheddable shell for the micelles when in reducing environment. The results showed that the dual responses to both acidic and reducing environment could enhance drug release, and it was found that the DOX-loaded micelles showed low cytotoxicity.

**Fig. 4:**
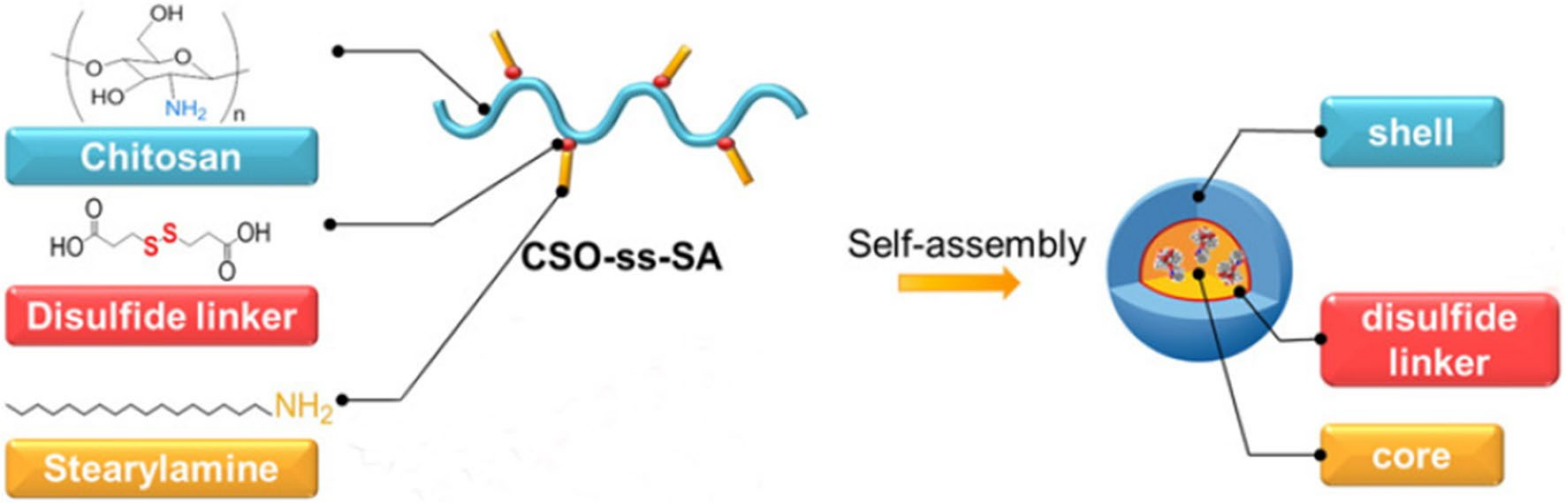
Schematic presentation of redox-responsive CSO-ss-SA micelles (Reprinted with permission from [35]. Copyright 2015 Elsevier)

Recently, it was reported that linking the hydrophobic structure of deoxycholic acid (DOCA) to the hydrophilic hyaluronic acid (HA) backbone via disulfide bonds can be used to synthesize the redox-responsive carrier HA-ss-DOCA for paclitaxel (PTX) targeted intracellular delivery [31]. The results showed that PTX-loaded HA-ss-DOCA micelles could release drugs rapidly inside the cells and the endocytosis mediated by HA-receptors could enhance the drug accumulation at tumor sites. There was another study introducing a chitosan based glycolipid-like nanocarrier (CSO-ss-SA) through the link between the hydrophobic structure and the hydrophilic backbone with disulfide bonds to realize the co-delivery of siRNA and drugs (Fig. 4) [32, 35]. In the study, the model drug Nile Red was released 8–11 h later than siRNA, which achieved the synergistic treatment of genes and drugs.

Connecting cisplatin to the PLG chains through disulfide bonds can be utilized to synthesize novel redox-responsive micelles consisting of polyethylene glycol-poly-(l-glutamic acid) (PEG-PLG) [33]. The micelles in this study showed well-controlled cisplatin loading yield, excellent redox-responsive drug release kinetics, stronger antitumor activity and less biological toxicity.

#### Disulfide bonds attached to the surface of nanoparticles

Nowadays the technology of nanoparticles is becoming more and more mature (Fig. 1c), with different nanoparticles studied extensively including magnetic nanoparticles (Fe_3_O_4_) [36], gold nanoparticles [37, 38], Ag nanoparticles [39], silica nanoparticles [40–44], gold/mesoporous silica hybrid nanoparticles (GoMe) [45], and mesoporous manganese silicate coated silica nanoparticles (MMSSN) [46]. Among them, mesoporous silica nanoparticles (MSN) have been well studied. Utilizing disulfide bonds to link modified structures to nanoparticles’ surface can enrich the function of nanoparticles [43], such as enhancing the redox-responsiveness and targeting capabilities [42]. In addition, disulfide bonds can be linked with genes or drugs [41], which can realize the rapid release of genes or drugs under reducing environments.

Han linked transferrin (Tf) as both a capping agent and a targeting group on the surface of MSNs via redox-responsive disulfide bonds, which could encapsulate doxorubicin (DOX) efficiently [42]. When the system is exposed to GSH, DOX can burst out. The system has excellent biocompatibility, accumulation in tumor cells and significant enhancement of targeting. In another study, disulfide bonds were utilized to attach siRNA to the surface of MSNs, which realized the co-delivery of DOX and siRNA [41]. The results showed that MSNs-SS-siRNA@Dox significantly enhanced the antitumor activity and achieved satisfactory therapeutic effects on tumor inhibition both in vitro and in vivo.

#### Disulfide bonds linking two moieties

Disulfide bonds can also link two moieties together even they are with different roles (Fig. 1d) [47], which is a good strategy to make the structure and function of carriers more abundant [48]. Linking two polymers which are hydrophilic and hydrophobic respectively via disulfide bonds can form an amphiphilic copolymer and then self-assemble into micelles [49–52], which can deliver hydrophobic drugs and improve their solubility.

**Fig. 5:**
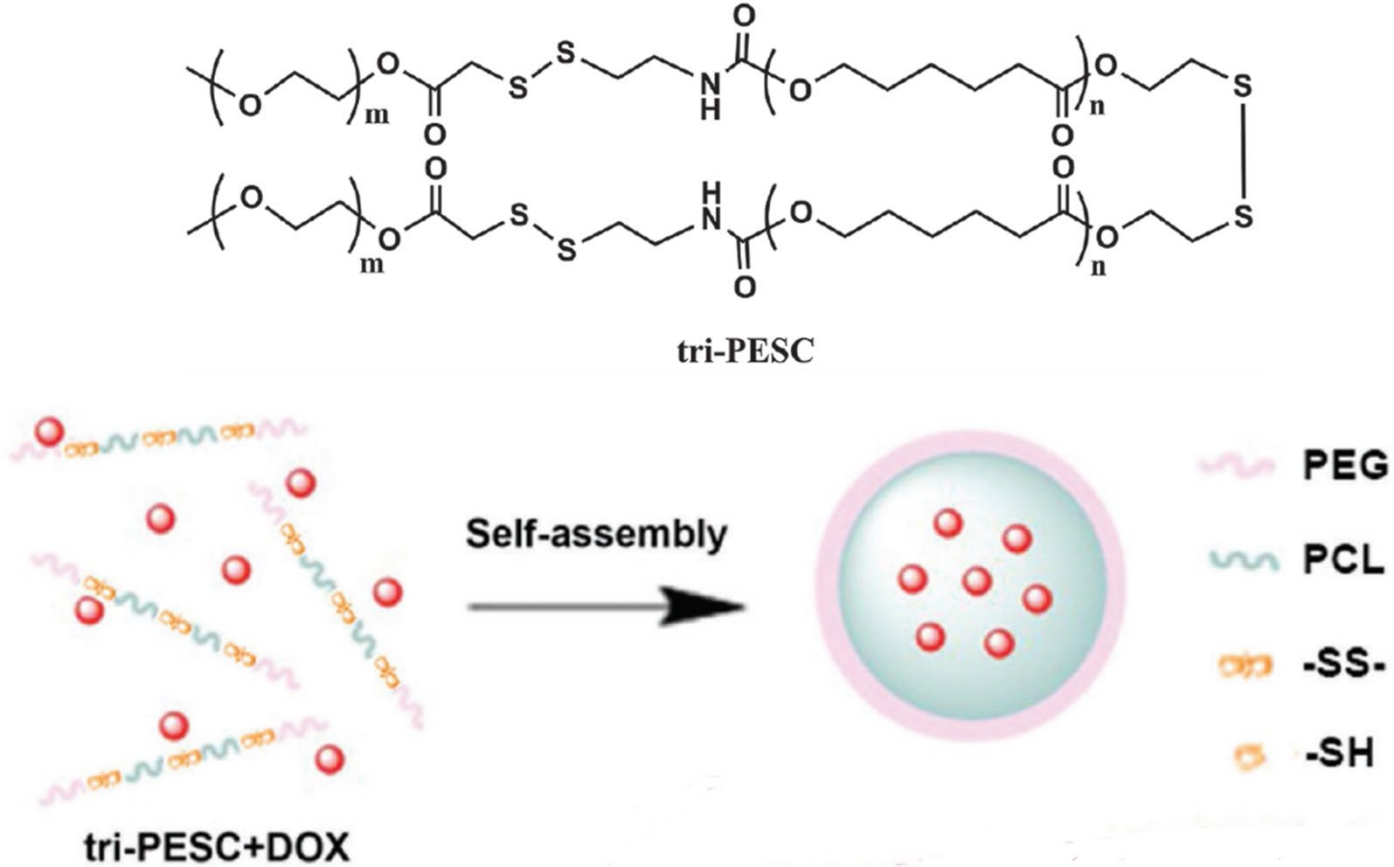
Schematic representation of redox-responsive tri-PESC micelles (Reprinted with permission from [47]. Copyright 2016 John Wiley and Sons)

Both the premature release of cargoes when the carrier is still in blood circulation and insufficient intracellular drug release are critical issues to be solved. Li et al. introduced three disulfide bonds into the amphiphilic poly(ethylene glycol)-polycaprolactone copolymer blocks to synthesize triple-sensitive cleavable polymeric nanocarrier mPEG-SS-PCL-SS-PCL-SS-mPEG) (tri-PESC), which was demonstrated to be a good way to improve the sensitivity to narrow the range of GSH concentration (Fig. 5) [47]. Tri-PESC NPs remain intact when in blood circulation due to stable disulfide bonds, whereas the loaded drugs are effectively released when encountering tumor cells with high GSH concentration. Another study used disulfide linkers to connect pH-responsive p(His)_n_ block with biocompatible phospholipid analog poly(2-methacryloyloxyethyl phosphorylcholine) [p(MPC)] block to create a pH/redox dual stimuli-responsive block copolymer [50]. The block copolymers are self-assembled into uniform micelles to encapsulate DOX effectively and enhance drug release and antitumor activity.

### Disulfide bonds acting as cross-linking agents

At present, micelles assembled by the amphiphilic diblock or triblock copolymer are extensively studied. Due to the complexity of in vivo environment, micelles often have a poor stability in the delivery process, which easily lead to the loss of drugs. Furthermore, unexpected side effects may be caused in this case. To solve this problem, crosslinked micelles are often used to enhance the stability and thus the loss of cargoes could be effectively prevented before reaching cells or other target sites. Meanwhile, the crosslinked structure may also act as barriers to drug release, slowing down the rate of release. Disulfide bonds exist mainly in the core–shell form of micelles as cross-linkers, such as shell crosslinked micelles (SCM, Fig. 1e) [53] and core crosslinked micelles (Fig. 1f) [54, 55]. With the rapid response of disulfide bonds to the reducing environment, the release of the drug can be promoted.

Lee et al. designed a redox-responsive shell-crosslinked micelle. Two low toxicity materials, poly-(ethylene glycol) (PEG) and polyamino acid, were chosen to self-assemble the triblock copolymer poly(ethylene glycol)-b-poly(l-lysine)-b-poly(l-phenylalanine) (PEG-b-PLys-b-Ppha) [53]. The use of disulfide cross-linking in the middle shell enhanced the stability of micelles. It was demonstrated that shell cross-linking could significantly improve the physical stability of micelles. With the increase of the degree of crosslinking, the polyplex can slow down the release of methotrexate (MTX) more effectively.

**Fig. 6:**
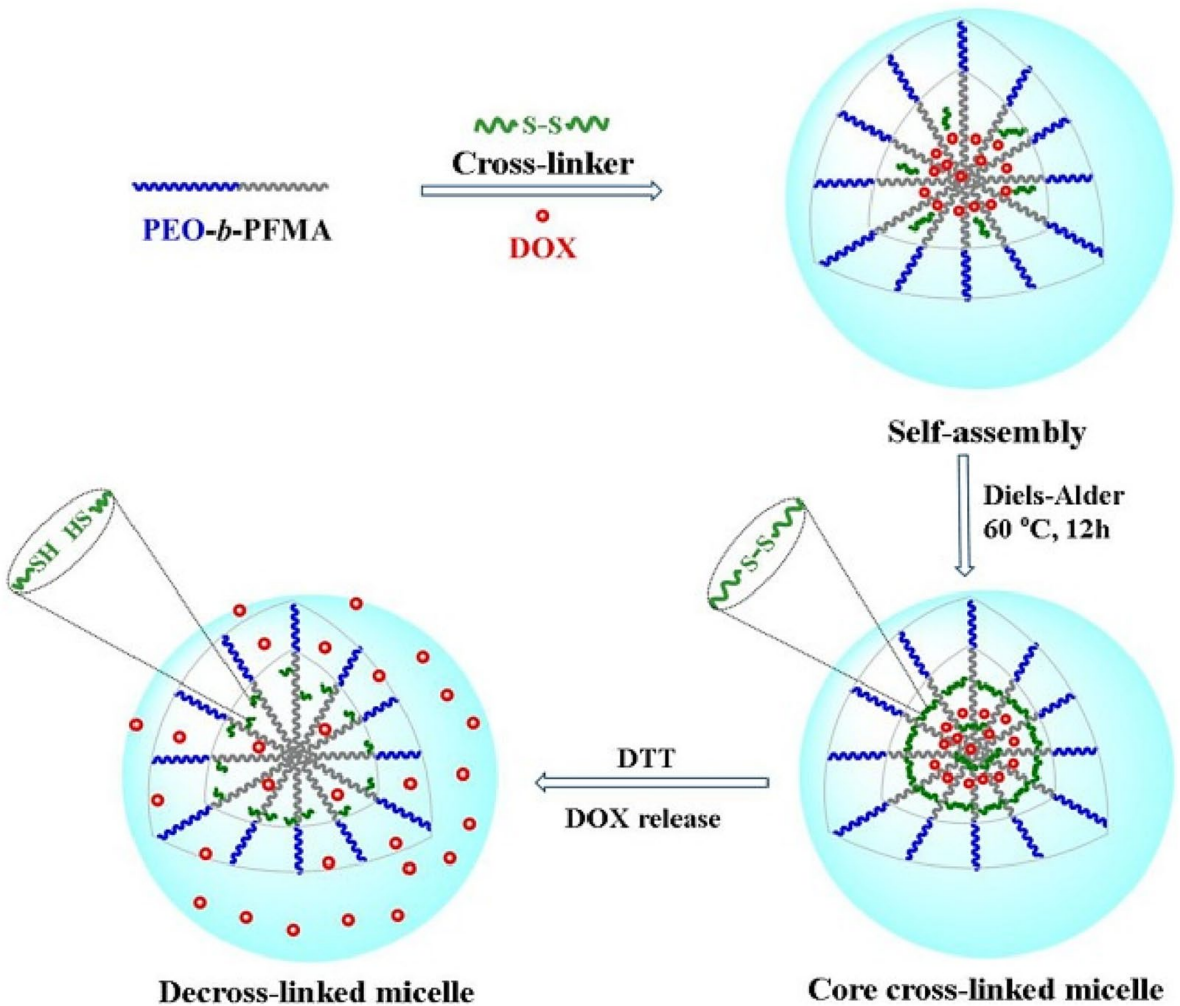
Schematic representation of the core crosslinked micelles and morphology transition under DTT reduction (Reprinted with permission from [54]. Copyright 2016 J John Wiley and Sons)

The Diels–Alder click-type reaction can be utilized to synthesize a redox-responsive core cross-linked micelles poly(ethylene oxide)-b-poly(furfuryl methacrylate) (PEO-b-PFMA), which encapsulated DOX in the hydrophobic core (Fig. 6) [54]. Results showed that the core cross-linked micelles could enhance the stability of the micelle under physiological conditions. While under the environment of DTT, the disulfide bonds rapidly broke down, which caused micellar dissociation and released DOX.

### Disulfide bonds in other redox-responsive delivery systems

There are some other redox-responsive delivery systems containing disulfide bonds, such as polymeric nanogel with disulfide linkages [56, 57], liposome [58, 59], dendrimer–drug conjugates with disulfide linkages [60] and other structures. However, currently no much work is done on this topic, and thus detailed applications are not discussed here.

## Conclusion

The reducing environment in tumor cells has appeared as a natural stimulus for effective intracellular delivery for many years. Redox-responsive drug delivery systems have been recognized as a valuable strategy to achieve efficient intracellular drug delivery with low cytotoxicity. Utilizing the differences between the special microenvironments of tumor cells and normal cells, redox-responsive delivery systems can meet the requirement of targeted therapy in theory. With various reducing structures or functional groups being added, the redox-responsive system is moving in the direction of low toxicity and high efficiency. However, some studies have reported that the reduction of thiolated polymers takes several hours under physiological reducing conditions, which may be a future barrier to overcome [61, 62].

In fact, there exists multiple unique stimuli in tumor microenvironment such as pH, enzymes, and oxidants, while many other external stimuli could also utilized including magnetic field, temperature, light and ultrasound [10, 63, 64]. In addition to the reduction property in tumor microenvironment, other features could be further developed like abundant enzymes in the lysosomes and slightly lower pH in the endosomes and lysosomes. The pH-responsive drug carriers can be degraded under endo/lysosomal pH conditions, which contributes to the effective drug release. However, the chemical cleavage of a pH-responsive substructure is not fast enough, resulting in slow response speed [62]. Recent studies have reported that tumor cells not only acidify but also alkalize, in which situation pH-responsive drug delivery systems do not apply [65, 66]. Enzyme-responsive drug delivery systems have been recognized as a valuable strategy to achieve efficient intracellular drug delivery [67, 68], as there are abundant enzymes including proteases, matrix metalloproteinases and hyaluronidases in the lysosomes [62]. Despite the fact that enzyme-responsive drug delivery systems have high specificity, the complicated synthesis methods and harsh lysosomal conditions leading to drug degradation limit their feasibility in application [14].

Therefore, the delivery systems which can respond to multiple stimuli have become a popular strategy of current research, because they are safer and more targeted than delivery systems with one stimulus [40, 43, 59, 69, 70]. The development of advanced gene/drug or multidrug codelivery systems with stimuli-responsive release manner for tumor therapy is also desirable [38, 71]. However, combining multiple functions into one delivery system remains a challenge. For example, in order to visualize drug delivery and release, combining imaging technique and therapy will facilitate the study of drug distribution and controlled release simultaneously [20], which is difficult to be achieved currently. Moreover, MDR (multi-drug resistance) leading to low clinical anticancer efficacy remains a complicated problem to be solved [34, 52, 72]. In addition, delivery systems with multiple functions while being simple in structure and delivery systems with specific structures while being less cost are often contradictory.

Even so, many novel intracellular environment-responsive drug delivery systems have been reported, and their structure and function are constantly being improved. It is believed that with the continuous development of materials science and tumor treatment, problems mentioned above can be effectively solved by the development of “smart” drug delivery systems such as the redox-responsive system.

## Abbreviations

ATNDDS: antitumor nano drug delivery systems
NADPH: nicotinamide adenine dinucleotide phosphate
GSH: glutathione
ROS: reactive oxygen species
MDR: multi-drug resistance
TMBQ: ‘‘trimethyl-locked’’ benzoquinone
SPDP: *N*-succinimidyl-3-(2-pyridyldithiol) propionate)
DSDMA: disulfide-based dimethacrylate
CBZ: *N*,*N*′-cystaminebisacrylamide
AEPZ: 1-(2-aminoethyl) piperazine
BPS: bioreducible polyspermine
PEI: polyetherimide
PSI: polysuccinimide
PEG: polyethylene glycol
HA: hydrophilic hyaluronic acid
DOCA: deoxycholic acid
PTX: paclitaxel
PEG-PLG: polyethylene glycol-poly-(l-glutamic acid)
Fe_3_O_4_: magnetic nanoparticles
GoMe: gold/mesoporous silica hybrid nanoparticles
MMSSN: mesoporous manganese silicate coated silica nanoparticles
MSN: mesoporous silica nanoparticles
Tf: transferrin
DOX: doxorubicin
tri-PESC: mPEG-SS-PCL-SS-PCL-SS-mPEG
RAFT: reversible addition-fragmentation chain transfer
ROP: ring-opening polymerization
p(MPC): poly(2-methacryloyloxyethyl phosphorylcholine)
SCM: shell crosslinked micelles
PEG-b-PLys-b-Ppha: poly(ethylene glycol)-b-poly(l-lysine)-b-poly(l-phenylalanine)
MTX: methotrexate
PEO-b-PFMA: poly(ethylene oxide)-b-poly(furfuryl methacrylate)
